# Medical, Sensorimotor and Cognitive Factors Associated With Gait Variability: A Longitudinal Population-Based Study

**DOI:** 10.3389/fnagi.2018.00419

**Published:** 2018-12-18

**Authors:** Oshadi Jayakody, Monique Breslin, Velandai Srikanth, Michele Callisaya

**Affiliations:** ^1^Menzies Institute for Medical Research, University of Tasmania, Hobart, TAS, Australia; ^2^Department of Medicine, Peninsula Health, Monash University, Melbourne, VIC, Australia

**Keywords:** gait, gait variability, longitudinal study, cognition, sensorimotor, older age

## Abstract

**Background:** Greater gait variability increases the risk of falls. However, little is known about changes in gait variability in older age. The aims of this study were to examine: (1) change in gait variability across time and (2) factors that predict overall mean gait variability and its change over time.

**Methods:** Participants (*n* = 410; mean age 72 years) were assessed at baseline and during follow up visits at an average of 30 and 54 months. Step time, step length, step width and double support time (DST) were measured using a GAITRite walkway. Variability was calculated as the standard deviation of all steps for each individual. Covariates included demographic, medical, sensorimotor and cognitive factors. Mixed models were used to determine (1) change in gait variability over time (2) factors that predicted or modified any change.

**Results:** Over 4.6 years the presence of cardiovascular disease at baseline increased the rate of change for step length variability (*p* = 0.04 for interaction), lower education increased the rate of change for DST variability (*p* = 0.04) and weaker quadriceps strength increased the rate of change for step width variability (*p* = 0.01). Greater postural sway predicted greater variability on average across the three phases (*p* < 0.05). Arthritis, a higher body mass index (BMI), slower processing speed and lower quadriceps strength predicted greater mean step time variability (*p* < 0.05). Arthritis and a higher BMI predicted greater mean step length variability, while slower processing speed and BMI predicted greater mean DST variability (*p* < 0.05).

**Conclusion:** Over a nearly 5-year period, variability in different gait measures do not show uniform changes over time. Furthermore, each variability measure appears to be modified and predicted by different factors. These results provide information on potential targets for future trials to maintain mobility and independence in older age.

## Introduction

An estimated 30–35% of adults aged 70 and older have abnormal gait (Verghese et al., 2006), increasing the risk of falls, hospitalization and institutionalization (Montero-Odasso et al., 2005; Verghese et al., 2006). Traditionally, changes in gait speed are used as markers of gait dysfunction. However, a growing body of literature has investigated intra-individual gait variability, the fluctuation in the value of a gait parameter from one step to the next (Callisaya et al., 2010). Gait variability is potentially a more sensitive predictor of adverse events such as falls (Brach et al., 2005; Verghese et al., 2009; Callisaya et al., 2011). The sheer increase in the global older population (Department of Economic and Social Affairs, 2015) and the high prevalence of gait impairments make understanding how gait variability changes in older age and what factors might predict this change an important topic for investigation.

Evidence on whether age is associated with gait variability is limited to cross-sectional studies. Most studies have compared gait variability between younger and older people (Gabell and Nayak, 1984; Hausdorff et al., 1997; Stolze et al., 2000; Grabiner et al., 2001; Menz et al., 2003; Owings and Grabiner, 2004a,b; Woledge et al., 2005; Kang and Dingwell, 2008; Beauchet et al., 2009), reporting either no age-related differences (Gabell and Nayak, 1984; Hausdorff et al., 1997), or greater variability in spatial (Grabiner et al., 2001; Owings and Grabiner, 2004a,b; Woledge et al., 2005; Kang and Dingwell, 2008; Beauchet et al., 2009) and temporal measures (Menz et al., 2003; Kang and Dingwell, 2008) in older age groups. In studies of just older people, advancing age was associated with greater spatial (Helbostad and Moe-Nilssen, 2003; Callisaya et al., 2010; Verlinden et al., 2013) and temporal variability (Hausdorff et al., 2001b; Callisaya et al., 2010; Verlinden et al., 2013; Kirkwood et al., 2016). However, the majority of studies are from small samples of volunteers or older adults from geriatric or rehabilitation clinics (Gabell and Nayak, 1984; Hausdorff et al., 1997; Stolze et al., 2000; Owings and Grabiner, 2004a; Woledge et al., 2005; Kang and Dingwell, 2008; Beauchet et al., 2009), limiting generalizability.

Longitudinal studies would assist in better understanding the role of aging on gait variability. Furthermore, despite cross-sectional associations between poorer physical (Hausdorff et al., 2001a; Brach et al., 2008a; Kang and Dingwell, 2008; Callisaya et al., 2009) and cognitive function (Holtzer et al., 2012b; Martin et al., 2012; Beauchet et al., 2014) with greater gait variability, no studies to our knowledge have examined the factors that may modify longitudinal changes in gait variability. Such information is clinically important in determining individuals at increased risk of declining gait and hence adverse outcomes such as falls.

Therefore, the aims of this study are in a population-based sample of older people: (1) to examine the longitudinal associations between age and a range of temporal and spatial gait variability measures; (2) to examine the demographic, medical, sensorimotor and cognitive factors that predict overall mean gait variability and its change over time.

## Materials and Methods

### Study Participants

**FIGURE 1:**
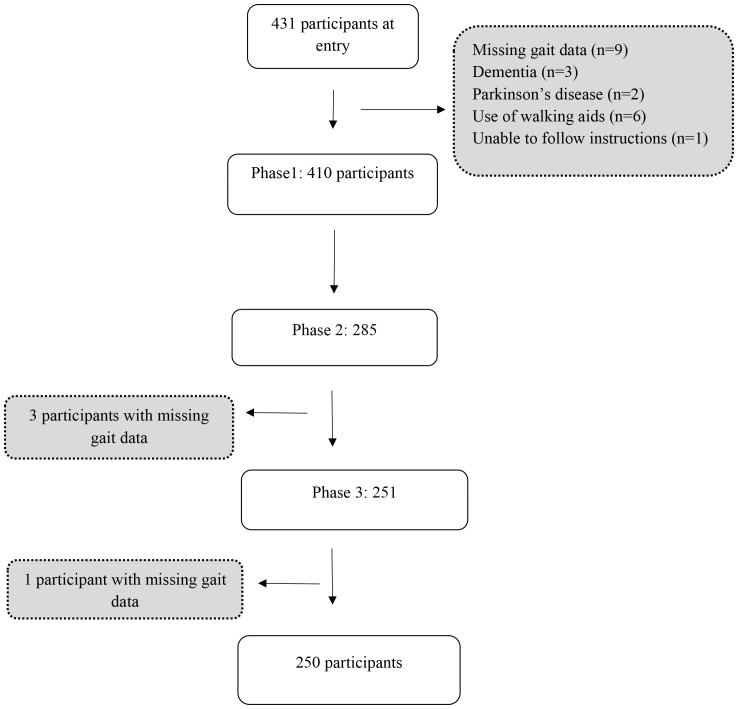
Study flow chart.

The Tasmanian Study of Cognition and Gait (TASCOG) is a population based longitudinal study of gait, cognition and brain imaging in older people. A community dwelling sample of adults aged between 60 and 85 years (*n* = 431) were randomly selected from the Southern Tasmanian electoral roll. Participants were excluded if they were institutionalized, or had any contraindication to MRI (a requirement of the parent study). Individuals were also excluded (Figure 1) if they had a history of dementia (*n* = 3), Parkinson’s disease (*n* = 2), missing gait data (*n* = 9), used a walking aid (*n* = 6), or were unable to follow simple commands in English (*n* = 1). Ethical clearance was obtained from the Southern Tasmanian Health and Medical Human Research Ethics Committee and written consent was obtained from all participants. The inception cohort, assembled from January 2005 (baseline), followed-up in March 2008 (phase 2) and March 2010 (phase 3) used identical methods.

### Gait Assessment

Spatial and temporal measures of gait were determined from the footfalls recorded on the GAITRite system, a 4.6 m computerized walkway, (GAITRite system, CIR Systems, Havertown, PA, United States), with excellent test–retest reliability (Menz et al., 2004). Each participant completed six walks at their preferred speed. To allow for acceleration and deceleration participants walked 2 m before and after the walkway. Gait speed (cm/s) was directly obtained from the GAITRite software and intra-individual variability of 2 spatial (step length, step width) and 2 temporal (step time, DST) gait measures were calculated as the standard deviations (SD) of all steps over the six walks as previously described (Callisaya et al., 2010). Gait variability is commonly quantified as either the SD or the coefficient of variation [CoV = (SD/mean) × 100]. Here we use the SD in order to report change in variability in each of the gait measures’ original units. Variability in these gait measures were selected as they have previously been found to be associated with advancing age (Kang and Dingwell, 2008; Beauchet et al., 2009; Callisaya et al., 2010) and risk of falls (Hausdorff et al., 2001b; Brach et al., 2005; Callisaya et al., 2011), and represent both spatial and temporal parameters in the sagittal and frontal planes (Callisaya et al., 2010; Martin et al., 2012). Variability in stance and swing time, although having been examined in prior cross sectional analyses (Hausdorff et al., 1997; Beauchet et al., 2009; Kirkwood et al., 2016), were not included due to high correlations with step time variability (stance time variability *r* = 0.86; swing time variability *r* = 0.80). Single support time was also not considered since it is the opposite of DST.

### Baseline Covariates

The following demographic, medical, sensorimotor and cognitive factors were assessed at baseline. Demographic variables included age, sex and level of education (summarized into a binary variable using high school level education and below as the cut off). Height (m) and weight (kg) were recorded to calculate the BMI.

#### Medical History

Presence of lower limb arthritis and CVD (hypertension, hypercholesterolemia, ischemic heart disease, stroke and diabetes mellitus) were recorded with a standardized questionnaire. CVDs were grouped into a summary binary variable based on the presence or absence of any CVD. Mood was assessed using the Geriatric Depression Scale (short version). Participants were classified as depressed, based on a score of >5.

#### Sensorimotor Factors

Postural sway, quadriceps strength, edge contrast sensitivity and proprioception were measured with the short form of Physiological Profile Assessment (Lord et al., 2003).

(1) *Postural sway*: measured on a foam mat for 30 s with the eyes open (EO) and eyes closed (EC) [sum of maximum medial-lateral and anterior-posterior sway (mm); no upper limit].

(2) *Quadriceps strength*: the maximal isometric quadriceps strength of the dominant leg (kg) measured in sitting (up to 100 kg; >30 kg is considered excellent).

(3) *Edge contrast sensitivity*: an indicator of visual contrast sensitivity [measured using the Melbourne edge test (dB); range 0–24].

(4) *Proprioception*: perception of joint and body segments or movement in the space (Sherrington, 1906) (measured with a lower limb matching task using a vertical clear acrylic sheet placed between the seated participant’s legs; no upper limit; values of <1 degree considered good).

(5) *Grip strength* was quantified with a bulb dynamometer as the average of two measurements of dominant and of non-dominant hand (pounds per square inch).

#### Cognitive Function

The following tests were used to measure cognition: (a) *Executive function*: the Controlled Word Association Test (as many words as possible in 1 min; using the letters F, A, and S), the Victoria Stroop test (to correct for processing speed the difference in time to compete Stroop color test– Stroop word test was used); (b) *Processing speed-attention:* the Symbol Search (range 0–60), Digit Span (range 0–16) and Digit Symbol Coding (range 0–133) of Wechsler Adult Intelligence Scale-III, (c) *Visuospatial function:* the Rey Complex Figure copy task (range 0–36) and (d) *Memory:* the Hopkins Verbal Learning Test—Revised [Immediate recall (range 0–36), delayed recall (range 0–12), recognition range (0–12)] and a 20 min delayed reproduction of the Rey Complex Figure copy task (range 0–36). Raw test scores were grouped and subjected to principal component analyses deriving summary components for domains of executive function, processing speed-attention, memory and visuospatial ability as previously described (Callisaya et al., 2015).

### Data Analysis

STATA (StataCorp LLC, College Station, TX, United States) version 15.0 was used in all the analyses.

#### Changes in Gait Variability Over Time

Longitudinal associations between time and gait variability were examined using linear mixed effects models. The residual distributions of the step time and DST conditional models, when assessed for normality, showed positive skewness. Therefore, step time variability was transformed using *1/Y^1^* and DST variability by *1/(Sqrt(Y)*, which were chosen based on the results of a likelihood maximization procedure (STATA boxcox). Variables were back transformed for presentation of results. The model building procedure was as follows. Models were firstly adjusted for a priori confounders baseline age, sex and education. Interaction terms between time and each baseline covariate were then tested in separate models to determine if the covariate modified changes in each gait variability measure over time. Next, each covariate was tested individually to determine whether they were associated with gait variability over time. Finally, we built models using the significant interactions and predictor covariates from individual models in a stepwise fashion, with variables retained only if they remained significant. Although not a major aim of this study, but to allow for comparisons with other studies, models were also built to assess longitudinal changes in gait speed and the other absolute gait measures.

There was considerable participant attrition between baseline and the follow-up phases. Linear mixed effects models are able to provide an unbiased estimate of the regression coefficients in the case of such attrition, provided the reasons for drop-out depend only on the observed data (Little, 1995). There was no reason to believe otherwise in the case of this data. Attrition was found to depend on some outcome measures at baseline, and provided the model is correctly specified in relation to these variables, estimates of coefficients are unbiased.

## Results

### Sample Characteristics

Table 1 summarizes participant characteristics (*n* = 410).

**Table 1:** Baseline characteristics of the study sample (*n* = 410).

Variable	Mean SD or Frequency %	
Age (y), mean, *SD*	72.0	7.0
Male, *n*, %	233	56.8
Greater than high school education, *n*, %	200	46.4
BMI [kg/m^2^], mean, *SD*	27.9	4.7
Self-reported medical history, *n* %		
Hypertension	203	49.5
High Cholesterol	176	42.9
Angina	56	13.7
Myocardial Infarction	58	14.2
Diabetes Mellitus	51	12.4
Stroke	36	8.8
Prevalence of any CVD, *n* %	288	70.4
Lower limb arthritis, *n* %	142	35.2
Depression, *n* %	37	9.0
Postural sway (EO) [mm], mean, *SD*	41.4	17.5
Postural sway (EC) [mm], mean, *SD*	77.8	38.4
Quadriceps strength [kg], mean, *SD*	31.8	12.2
Proprioception [degrees], mean, *SD*	2.7	1.9
Edge contrast sensitivity [dB], mean, *SD*	20.1	2.4
Grip strength [psi], mean, *SD*	29.3	9.2
Gait characteristics, mean *SD*		
Gait speed [cm/s]	113.8	21.2
Step length variability [cm]	2.7	1.0
Double support time variability [ms]	20.7	10.6
Step width variability [cm]	2.1	0.7
Step time variability [ms]	22.2	13.2
Cognitive tests		
COWAT (words/min)	35.8	13.3
Stroop dots (seconds)	15.9	5.0
Stroop words (seconds)	21.6	8.1
Stroop colors (seconds)	35.8	13.3
Digit span (number correct)	15.8	3.8
Digit symbol coding (number correct)	49.6	15.2
Symbol search (number correct)	22.4	7.8
Hopkins immediate recall (number correct)	21.8	6.1
Hopkins delayed recall (number correct)	7.5	3.1
Hopkins recognition (number correct)	9.9	2.0
Rey complex figure copy (number correct)	31.9	4.9
Rey complex figure delay (number correct)	14.7	6.9


*This table summarizes the demographic, medical, sensorimotor, gait and cognitive characters of the sample at baseline*.

*SD, standard deviation; BMI, Body Mass Index; CVD, cardiovascular disease; EO, eyes open; EC, eyes closed; COWAT, Controlled Oral Word Association Test; mm, millimeter; kg, kilograms; ms, milliseconds; cm, centimeter; dB, decibel; psi, pounds per square inch*.

### Longitudinal Associations of Gait Variability Over Time

Significant increases, independent of baseline age, sex and level of education, were seen in step length variability (β 0.028 95%CI 0.004 to 0.052; *p* = 0.02), resulting in an increase of 0.14 cm over 5 years and in DST variability (β 0.223 95%CI 0.091 to 0.355; *p* = 0.001), indicating an increase of 1.12 ms over 5 years. The findings for variability of step time (β 0.085 95%CI -0.039 to 0.208; *p* = 0.18, corresponding to an increase of 0.43 ms over 5 years) and step width (β -0.001 95%CI -0.018 to 0.017; *p* = 0.94, a decrease of 0.005 cm over 5 years) were non-significant.

### Modifiers and Predictors of Gait Variability

For step length variability the interaction between CVD and time was significant (*p*-value for interaction = 0.03), indicating greater increases over time in the presence of baseline CVD. For DST variability the interaction between education and time was significant (*p* = 0.01), indicating greater increases over time in people with lower levels of education. Although step width variability did not increase over time on average, the interaction between quadriceps strength and time was significant (*p* = 0.02), indicating greater increases in those with weaker quadriceps muscles. None of the interactions for step time variability were significant (*p* > 0.05). Table 2 shows the effect of each of these interactions on the time coefficient. Table 3 shows the associations between factors tested one at a time with gait variability, adjusted for time terms and *a priori* confounders (age, sex, and education).

**Table 2:** Longitudinal changes of gait variability over time (*n* = 410).

	Step length variability, cm		DST variability, ms		Step width variability, cm		Step time variability, ms	
	β	95%CI	β	95%CI	β	95%CI	β	95%CI
Total change over time, per year:							0.085	-0.039, 0.208
No CVD	-0.009	-0.050, 0.033						
Presence CVD	0.046	0.017, 0.075						
Low education			0.431	0.233, 0.629				
High education			0.048	-0.175, 0.271				
25%^∗^ of quad strength					0.012	-0.010, 0.033		
50%^∗^ of quad strength					-0.002	-0.019, 0.016		
75%^∗^ of quad strength					-0.016	-0.037, 0.004		


*This table shows changes in individual gait variability measures per year; any change modified by demographic, medical, cognitive or sensorimotor factors is shown by presenting change over time at high and low values of the baseline covariate.*

*All models were adjusted for age, sex, and education; due to interactions with time, change depends on CVD status, educational level or quadriceps strength depending on the variability measure. No other interactions effects were significant and are therefore not presented. CVD, cardiovascular disease; kg, kilograms; quad, quadriceps;^∗^ meaning the 25th, 50th, and 75th percentiles of quadriceps strength.*

**Table 3:** Associations between medical, sensorimotor and cognitive factors with gait variability in individual models (*n* = 410); the effect of each baseline covariate on the average of gait measures over 3 phases.

	Step length variability, cm		DST variability, ms		Step width variability, cm		Step time variability, ms	
	β	95%CI	β	95%CI	β	95%CI	β	95%CI
CVD			0.153	-0.626, 0.932	0.111	-0.000, 0.223	0.399	-0.406, 1.203
Arthritis	0.228	**0.075, 0.381**	**1.289**	**0.487, 2.091**	-0.026	-0.138, 0.085	**2.439**	**1.526, 3.352**
BMI [kg/m^2^]	**0.020**	**0.004, 0.036**	**0.149**	**0.076, 0.221**	-0.005	-0.016, 0.006	**0.106**	**0.031, 0.181**
Postural sway (EC) [mm]	**0.003**	**0.002, 0.005**	**0.013**	**0.004, 0.022**	**0.002**	**0.001, 0.003**	**0.022**	**0.012, 0.031**
Postural sway (EO) [mm]	0.001	-0.001, 0.004	-0.001	-0.017, 0.014	0.001	-0.001, 0.003	0.007	-0.008, 0.022
Quadriceps strength [kg]	-**0.008**	-**0.014,**-**0.001**	-**0.045**	-**0.082,**-**0.008**			-**0.071**	-**0.112,**-**0.031**
Proprioception [degrees]	**0.065**	**0.038, 0.092**	**0.394**	**0.227, 0.562**	**0.025**	**0.006, 0.044**	**0.306**	**0.145, 0.468**
Edge contrast sensitivity [dB]	-**0.050**	-**0.075,**-**0.026**	-**0.165**	-**0.299,**-**0.030**	-0.013	-0.030, 0.004	-**0.191**	-**0.324,**-**0.058**
Grip strength [kg]	0.005	-0.018, 0.028	-0.057	-0.140, 0.026	-0.008	-0.025, 0.008	-0.072	-0.132, -0.012
Depression	0.068	-0.211, 0.346	0.237	-1.129, 1.603	0.157	-0.034, 0.349	1.169	-0.368, 2.706
Executive function	0.053	-0.020, 0.125	**0.472**	**0.105, 0.839**	0.007	-0.044, 0.058	**0.501**	**0.122, 0.879**
Processing speed-attention	-**0.062**	-**0.121,**-**0.003**	-**0.504**	-**0.791,**-**0.217**	-0.019	-0.060, 0.022	-**0.761**	-**1.066,**-**0.457**
Memory	0.002	-0.054, 0.058	-**0.301**	-**0.572,**-**0.031**	0.032	-0.007, 0.071	-**0.414**	-**0.686,**-**0.142**
Visuospatial function	-0.014	-0.031, 0.002	-**0.100**	-**0.183,**-**0.017**	-0.004	-0.016, 0.008	-**0.129**	-**0.216,**-**0.042**


*Models are adjusted for age, sex, and education, CVD × time (models for step length variability) and education × time (DST variability) and quadriceps strength × time (models for step width variability); higher scores on executive function indicate worse performance, whereas higher scores indicate better performance for other cognitive tests; CVD, cardiovascular disease; EO, eyes open; EC, eyes closed; BMI, Body Mass Index; kg, kilograms; cm, centimeter; mm, millimeter; dB, decibel. The associations that are significant (p < 0.05) are in bold.*

Table 4 shows final models for the four gait variability outcomes. The model for step length variability included a CVD × time term (*p* = 0.04), arthritis (*p* = 0.01), postural sway (EC) (*p* < 0.001) and BMI (*p* = 0.04). The model for DST variability included an education × time term (*p* = 0.04), postural sway (EC) (*p* = 0.002), BMI (*p* < 0.001) and processing speed (*p* < 0.003). The final model for step width variability included a quadriceps strength × time term (*p* = 0.01) and postural sway (EC) (*p* < 0.001). Although step time variability did not change over time, greater BMI (*p* = 0.03), arthritis (*p* < 0.001), lower quadriceps strength (*p* = 0.02), greater postural sway (EC) (*p* < 0.001) and slower processing speed at baseline (*p* < 0.001) were associated with greater mean variability over the three phases.

**Table 4:** Associations between medical, sensorimotor and cognitive factors with gait variability in final models (*n* = 410).

	Step length variability, cm		DST variability, ms		Step width variability, cm		Step time variability, ms	
	β	95%CI	β	95%CI	β	95%CI	β	95%CI
Age	0.017	**0.006, 0.028**	**0.125**	**0.072, 0.178**	**0.012**	**0.004, 0.020**	**0.073**	**0.013, 0.133**
Male	**0.180**	**0.038, 0.322**	-**1.361**	-**2.138,**-**0.585**	**0.161**	**0.043, 0.279**	0.516	-0.359, 1.392
Education	0.039	-0.102, 0.180			0.026	-0.078, 0.130	0.642	-0.193, 1.477
Total effect of time:							0.053	-0.091, 0.198
No CVD	-0.009	-0.049, 0.032						
Presence CVD	**0.045**	**0.016, 0.074**						
Low education			**0.424**	**0.216, 0.632**				
High education			0.101	-0.138, 0.339				
25%^∗^ of quad strength					0.015	-0.007, 0.037		
50%^∗^ of quad strength					0.001	-0.017, 0.018		
75%^∗^ of quad strength					-0.015	-0.036, 0.005		
BMI [kg/m^2^]	**0.017**	**0.001, 0.032**	**0.167**	**0.089, 0.246**			**0.099**	**0.012, 0.186**
Postural sway (EC) [mm]	**0.003**	**0.002, 0.005**	**0.014**	**0.004, 0.022**	**0.002**	**0.001, 0.003**	**0.019**	**0.010, 0.029**
Quadriceps strength							-**0.046**	-**0.085,**-**0.006**
Processing speed-attention			-**0.429**	-**0.710,**-**0.147**			-**0.585**	-**0.896,**-**0.274**
Arthritis	**0.202**	**0.051, 0.353**					**1.901**	**0.886, 2.916**


*Final models show the associations baseline covariates with gait variability in the presence of all significant modifiers and predictors.*

*CVD, cardiovascular disease; EC, eyes closed; BMI, Body Mass Index; quad, quadriceps; kg, kilograms; cm, centimeter; mm, millimeter.*

*^∗^Meaning the 25th, 50th, and 75th percentiles of quadriceps strength. The associations that are significant (p < 0.05) are in bold.*

### Longitudinal Changes and Factors Associated With Gait Speed and Other Temporal and Spatial Measures

Gait speed significantly decreased over time independent of baseline age, sex and level of education (β -1.159 95%CI -1.502 to -0.816; *p* < 0.001). Step length shortened (β -0.685 95%CI -0.811 to -0.560; *p* < 0.001), while DST (β 6.582 95%CI 5.285 to 7.879; *p* < 0.001) and base of support (β 0.139 95%CI 0.097 to -0.182; *p* < 0.001) significantly increased over time. Step time did not change (β -0.196 95%CI -1.025 to 0.632; *p* = 0.642) over the 4.6 years. The results of the final models for gait speed and the other absolute gait measures are presented in Supplementary Table S1.

## Discussion

This is the first study, to our knowledge, to undertake a longitudinal analysis of gait variability in a population-based sample of older people. Greater increases in variability were seen in people with CVD at baseline for step length, low levels of education for DST, and those with weaker quadriceps strength for step width. Furthermore, a number of baseline factors were associated with higher variability on average over the 3 phases. Greater postural sway (EC), BMI and arthritis predicted higher step length variability. Greater postural sway (EC), BMI and slower processing speed predicted higher DST variability. Greater postural sway (EC) predicted greater step width variability. Although step time variability did not increase over time, greater postural sway (EC), greater BMI, arthritis, lower quadriceps strength and slower processing speed predicted greater variability across the three phases. These findings increase knowledge on how gait variability changes in older age and assist in identifying factors that may be developed into strategies to prevent gait impairments among older people.

Few studies have examined the longitudinal changes in intra-individual gait variability. Prior studies have been in small samples of participants with specific diagnoses such as Alzheimer’s disease (Wittwer et al., 2010), subcortical vascular encephalopathy (Bäzner et al., 2000) and Huntington’s Disease (Rao et al., 2011), and report increases in stride length (Rao et al., 2011; Wittwer et al., 2010) and temporal variability measures (Bäzner et al., 2000; Rao et al., 2011). In our community-based cohort of people without dementia, changes over time were not consistent over the different temporal and spatial measures. Consistent with prior cross-sectional studies, step length (Callisaya et al., 2010; Verlinden et al., 2013) and DST variability (Callisaya et al., 2010; Verlinden et al., 2013) increased over time. This is important as we previously found that step length and DST variability measures, but not step width or step time variability, were linearly associated with increased risk of falls over a 12 months period (Callisaya et al., 2011).

Importantly, factors were identified that modified these associations, and that of step width variability, over time. Those with CVD had greater increases in step length variability. CVD is linked to vascular changes in the brain (i.e., white matter hyperintensities and brain infarcts), that can cause impairments in both cognition (De Groot et al., 2000; Wang et al., 2016) and gait (Rosano et al., 2007; Callisaya et al., 2013; Wang et al., 2016) even in people without dementia. Furthermore CVD, particularly diabetes mellitus, might disrupt peripheral sensorimotor abilities (i.e., lower extremity sensation and vision) (Brach et al., 2008b), resulting in increased step length variability over time. Therefore, the impact of CVD on central (i.e., disruption of pathways important for attention and motor control) and peripheral mechanisms (i.e., sensory loss) may disrupt dynamic balance resulting in the need to alter step length to maintain postural control whilst walking. These findings offer potential avenues of preventing increased gait variability in older age via controlling the advancement of CVD. Lower education levels accelerated increases in DST variability over time. Education is a known proxy for cognitive reserve (Stern, 2009). People with greater cognitive reserve are known to cope better with either age or pathology related changes in the brain (Stern, 2009), opening up the possibility that they are also better able to compensate for brain changes involving gait control (Holtzer et al., 2012a). Cognitive reserve is developed via lifetime exposure to cognitively stimulating experience (i.e., education, occupation, leisure, and physical activity) (Stern, 2009), thus enhancing these factors throughout life may assist in maintaining better gait control in older age. The significant interaction effect between quadriceps strength and time suggested those with weaker quadriceps had greater increases in step width variability over time. Greater step width variability has cross-sectionally been associated with advancing age (Helbostad and Moe-Nilssen, 2003; Callisaya et al., 2010; Verlinden et al., 2013), and falls (Brach et al., 2005). Our findings suggest that muscle strengthening may be an important target for clinical trials aimed at preventing increases in step width variability over time. We found no change for step time variability over time. A potential explanation may be that both high and low step time variability have been found to be associated with falls (Callisaya et al., 2011), suggesting that perhaps age-related impairments may result in either high or low variability, canceling out any directional change of effect. In summary it appears variability in different gait characteristics do not show uniform age-related changes over time.

Although not modifying change over time, greater postural sway (EC), higher BMI (except for step width variability), arthritis (step length and step time variability), slower processing speed (DST and step time variability) and lower quadriceps strength (step time variability) were associated with greater variability on average over the 3 phases. Postural sway on a foam mat (EC) is a measure of balance and vestibular ability. Walking is a complex balance activity (Woledge et al., 2005) and may require a trade-off in the consistency of timing and length of steps in the presence of poorer balance (Callisaya et al., 2009). Arthritis may increase step length variability through increased pain, stiffness (Kang and Dingwell, 2008), reduced strength (Kang and Dingwell, 2008) or balance (Callisaya et al., 2009). Our findings were independent of strength and balance, suggesting that these other impairments may be at play. A potential reason for the associations between BMI and greater variability may be that the body’s fat distribution affects balance. However, in our study BMI was a predictor of greater step length, DST and step time variability independent of postural sway, suggesting other mechanisms such as cerebro- (Rosano et al., 2007) or peripheral-vascular disease might be important (Forhan and Gill, 2013). Similar to prior cross-sectional studies, slower processing speed was associated with higher temporal variability (Brach et al., 2008a), but not spatial measures. It is possible that processing speed and temporal variability measures (both related to timing) may have similar underlying neural mechanisms. Atrophy in widespread brain networks (Blumen et al., 2018), as well as white matter hyperintensities (De Groot et al., 2000) and subcortical infarcts (Baune et al., 2009) that may disrupt white matter fibers are associated with processing speed and are also likely to be important for the co-ordination of a consistent gait pattern (Srikanth et al., 2010; Blumen et al., 2018). However, we were unable to determine whether central slowing of processing speed disrupted the timing of gait, or that of peripheral slowing, with both likely to lead to poorer gait control.

Although not a main aim of this study, similar to prior studies we observed that gait speed slowed over time (Atkinson et al., 2007; Callisaya et al., 2013) and this was greater in the presence of arthritis and poorer proprioception. Changes in other absolute gait measures were associated with a multitude of covariates (Supplementary Table S1), but these were different from the covariates that modified or predicted the same measures change in variability. For example, greater increases in step length variability over time occurred in the presence of CVD, whereas greater decreases in absolute step length over time occurred in the presence of greater baseline age, arthritis and poorer proprioception. Interestingly, this suggests that absolute and variability measures may represent different constructs in gait.

Our study has a number of strengths. It is one of the only longitudinal studies in the context of gait variability over time, with 4.6 years of follow up. Our sample was randomly selected from the electoral roll, increasing generalizability to the wider community compared to studies of people with specific diseases (i.e., Alzheimer’s disease). Gait variability is multifaceted, and we studied a range of temporal and spatial measures in both the sagittal and frontal planes. Further, we carefully controlled for confounders, examined for interactions and built our models by examining the effect of each variable one by one. However, there are a few limitations to be noted. Gait assessment was conducted in an indoor environment, thus gait variability could differ from outdoor walking. We collected data over 27 mean steps (baseline), where some have suggested a minimum of 400 steps are required to determine gait variability (Owings and Grabiner, 2003). However, we carefully considered this and balanced it with unnecessary fatigue. A diagnosis of dementia was by self-report and it is therefore possible that our sample had undiagnosed dementia. Finally, we have a moderate level of participants lost to follow up (39%) which is not uncommon given the longitudinal nature of our study. The use of a mixed effects model means that baseline data for those lost to follow up was able to contribute to the analysis.

## Conclusion

Variability in DST, step length and step width increased over time, but only in those with lower educational levels, CVD presence of CVD and weak quadriceps, respectively. In addition, a range of musculoskeletal, cognitive and sensorimotor factors were found to predict greater variability across the three phases. These results provide important information on targets for future clinical trials to maintain mobility and independence in older age.

## Availability of Data and Material

The raw data supporting the conclusions of this manuscript will be made available by the corresponding author, without undue reservation, on reasonable request.

## Author Contributions

OJ analyzed and interpreted the data, and wrote the manuscript. MB performed the statistical analysis, involved in drafting the manuscript, and critically revised the manuscript. VS primary investigator of the study, involved in drafting the manuscript and critically revised the manuscript. MC analyzed and interpreted the data, a major contributor in writing the manuscript, and provided important intellectual content. All authors read and approved the final manuscript.

## Conflict of Interest Statement

The authors declare that the research was conducted in the absence of any commercial or financial relationships that could be construed as a potential conflict of interest. The handling Editor declared a past co-authorship with two of the authors MC and VS.

## Abbreviations

BMI: body mass index
CVD: cardiovascular disease
DST: double support time
Postural sway (EC): postural sway eyes closed
Postural sway (EO): postural sway eyes open
