# Supplementary material for: Medical, Sensorimotor and Cognitive Factors Associated With Gait Variability: A Longitudinal Population-Based Study
Source: Front Aging Neurosci. 2018 Dec 18;10:419. doi: 10.3389/fnagi.2018.00419 (PMC6305368; doi:10.3389/fnagi.2018.00419)
Supplement: Supplementary file 1 [file Table_1.DOCX]

**Supplementary table 1** Demographic, medical, cognitive and sensorimotor factors associated with change in absolute gait measures in final models

|  | **Gait speed (cm/s)** | | **DST (ms)** | | **Step length (cm)** | | **Step width (cm)** | | **Step time (ms)** | |
| --- | --- | --- | --- | --- | --- | --- | --- | --- | --- | --- |
|  | **β** | **95%CI** | **β** | **95%CI** | **β** | **95%CI** | **β** | **95%CI** | **β** | **95%CI** |
| Time | 0.418 | -0.430,1.266 | 9.349 | 4.660,14.037 | -0.058 | -0.364,0.249 | 0.054 | -0.030,0.138 | -1.714 | -3.309,-0.119 |
| Age | -1.176 | -1.445,-0.907 | 2.172 | 1.356, 2.988 | -0.482 | -0.593,-0.371 | 0.050 | 0.008, 0.092 | 0.978 | 0.271, 1.686 |
| Sex | 5.155 | 1.708, 8.602 | 2.942 | -8.131,14.015 | 7.459 | 5.984, 8.934 | 2.108 | 1.583, 2.634 | 34.137 | 25.210, 43.064 |
| Education | 1.091 | -2.394, 4.577 | -6.221 | -16.510, 4.067 | 0.410 | -0.999, 1.818 | 0.278 | -0.256, 0.812 | 1.045 | -8.120, 10.211 |
| Age×time | -0.070 | -0.123,-0.018 |  |  | -0.033 | -0.052,-0.014 | 0.011 | 0.005,0.018 | 0.134 | 0.007, 0.261 |
| Arthritis×time | -0.760 | -1.490,-0.030 |  |  | -0.343 | -0.609,-0.076 |  |  |  |  |
| Proprioception×time | -0.264 | -0.521,-0.007 | 1.751 | 0.837, 2.665 | -0.110 | -0.203,-0.017 |  |  |  |  |
| Grip ×time |  |  | -0.495 | -0.882,-0.109 |  |  |  |  |  |  |
| Depression×time |  |  | -8.630 | -14.265,-2.996 |  |  |  |  |  |  |
| Executive function×time |  |  |  |  |  |  | 0.054 | 0.010, 0.098 |  |  |
| Arthritis | -8.140 | -11.947,-4.332 | 18.396 | 7.610, 29.182 | -3.122 | -4.652,-1.591 |  |  | 10.528 | 1.177, 19.879 |
| BMI [kg/m^2^] | -0.954 | -1.334,-0.575 | 4.324 | 3.212, 5.437 | -0.491 | -0.645,-0.338 | 0.206 | 0.150,0.262 |  |  |
| Visuospatial function | 0.669 | 0.303, 1.035 |  |  | 0.226 | 0.072, 0.380 |  |  |  |  |
| Processing speed |  |  | -5.389 | -9.346, 1.431 |  |  | -0.285 | -0.516, -0.054 | -4.371 | -7.879,-0.864 |
| Memory |  |  |  |  | 0.716 | 0.186 , 1.247 |  |  |  |  |
| Executive function |  |  |  |  |  |  | 0.015 | -0.264,0.295 |  |  |
| Depression |  |  | 35.878 | 13.517, 58.240 |  |  |  |  |  |  |
| Edge contrast sensitivity [dB] |  |  |  |  |  |  |  |  | -1.118 | -2.043,-0.193 |
| Proprioception [degrees] |  |  | -7.072 | -9.698,-4.445 |  |  | -0.074 | -0.124, -0.024 |  |  |
| Grip [psi] |  |  | 1.830 | 0.217, 3.444 |  |  |  |  |  |  |

Models are adjusted for age, sex and education where appropriate; Abbreviations: DST, Double support time; BMI, Body Mass Index; kg, kilograms; cm, centimeter; mm, millimeter; dB, decibel; psi, pounds per square inch;
